# The Importance of Podocyte Adhesion for a Healthy Glomerulus

**DOI:** 10.3389/fendo.2014.00160

**Published:** 2014-10-14

**Authors:** Rachel Lennon, Michael J. Randles, Martin J. Humphries

**Affiliations:** ^1^Wellcome Trust Centre for Cell-Matrix Research, Faculty of Life Sciences, The University of Manchester, Manchester, UK; ^2^Institute of Human Development, Faculty of Medical and Human Sciences, The University of Manchester, Manchester, UK; ^3^Department of Paediatric Nephrology, Manchester Academic Health Science Centre, Central Manchester University Hospitals NHS Foundation Trust, Manchester, UK

**Keywords:** podocyte, adhesion and signaling molecules, cell junction, extracellular matrix, nephrotic syndrome

## Abstract

Podocytes are specialized epithelial cells that cover the outer surfaces of glomerular capillaries. Unique cell junctions, known as slit diaphragms, which feature nephrin and Neph family proteins in addition to components of adherens, tight, and gap junctions, connect adjacent podocyte foot processes. Single gene disorders affecting the slit diaphragm result in nephrotic syndrome in humans, characterized by massive loss of protein across the capillary wall. In addition to specialized cell junctions, interconnecting podocytes also adhere to the glomerular basement membrane (GBM) of the capillary wall. The GBM is a dense network of secreted, extracellular matrix (ECM) components and contains tissue-restricted isoforms of collagen IV and laminin in addition to other structural proteins and ECM regulators such as proteases and growth factors. The specialized niche of the GBM provides a scaffold for endothelial cells and podocytes to support their unique functions and human genetic mutations in GBM components lead to renal failure, thus highlighting the importance of cell–matrix interactions in the glomerulus. Cells adhere to ECM via adhesion receptors, including integrins, syndecans, and dystroglycan and in particular the integrin heterodimer α3β1 is required to maintain barrier integrity. Therefore, the sophisticated function of glomerular filtration relies on podocyte adhesion both at cell junctions and at the interface with the ECM. In health, the podocyte coordinates signals from cell junctions and cell–matrix interactions, in response to environmental cues in order to regulate filtration and as our understanding of mechanisms that control cell adhesion in the glomerulus develops, then insight into the effects of disease will improve. The ultimate goal will be to develop targeted therapies to prevent or repair defects in the filtration barrier and to restore glomerular function.

## Introduction

**Figure 1:**
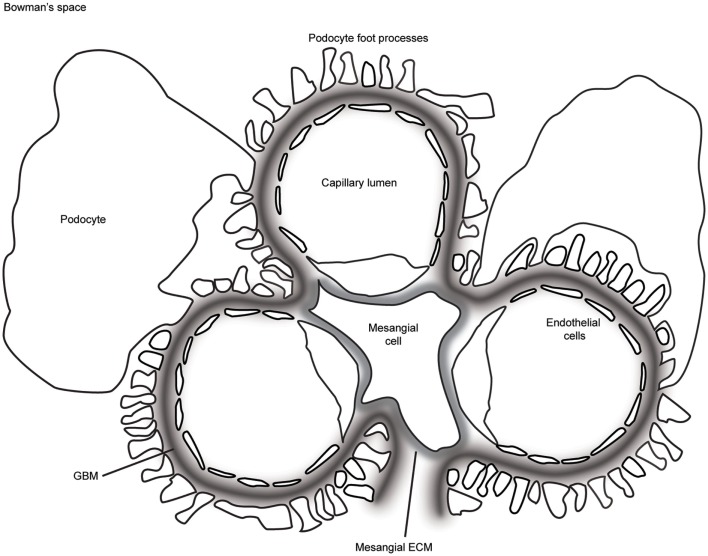
**A schematic representation of the glomerulus in cross-section**. The glomerular capillaries are lined on the inside with fenestrated endothelial cells, which are attached to the glomerular basement membrane (GBM). Podocytes cover the outer aspect of GBM with large cell bodies and inter-digitating foot processes. Mesangial cells and their associated extracellular matrix (ECM) connect adjacent capillaries, and the capillary bundle is contained within Bowman’s capsule.

The glomerulus is a highly sophisticated organelle that performs selective filtration of circulating blood. With a diameter of between 110 and 280 μm in humans (1), the glomerulus is a spherical bundle of capillaries contained by a cellular Bowman’s capsule. The capillaries are lined by fenestrated endothelial cells and covered by specialized epithelial cells known as podocytes (Figure 1). Between the cell layers, there is a thick glomerular basement membrane (GBM) providing a structural scaffold to support the capillary wall. Endothelial cells and their associated glycocalyx (2), the GBM, and podocytes together form the glomerular filtration barrier, which allows free permeability to water and small solutes but prevents the loss of macromolecules or cells from the blood into the primary filtrate. Each human kidney contains approximately 1 million glomeruli, and they perform this selective filtration to generate a remarkable 180 l of filtrate per day (3).

**Figure 2:**
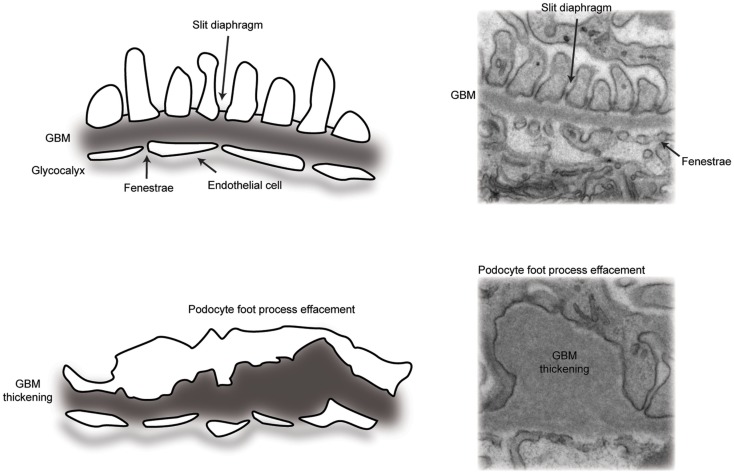
**A schematic representation of the glomerular filtration barrier**. (Top panel) transmission electron micrograph and schematic of the normal architecture of the multi-layered glomerular filtration barrier. (Bottom panel) transmission electron micrograph and schematic of glomerular filtration barrier defects, including loss of slit diaphragms and podocyte foot process effacement, in addition to thickening of the glomerular basement membrane.

Glomerular disease is characterized by reduced barrier integrity with consequent loss into the urine of protein (proteinuria) and or blood cells (hematuria). Barrier dysfunction is characterized by flattening or effacement of podocyte foot processes, as visualized by electron microscopy (Figure 2). Causes of barrier disruption range from congenital disorders associated with genetic mutations to acquired disease linked to a range of inflammatory or metabolic disturbances, which may specifically target the glomerulus or be part of a wider systemic illness, such as diabetes mellitus. Persistent glomerular dysfunction with proteinuria leads to chronic and ultimately end-stage kidney disease with a rapidly accelerating impact on worldwide healthcare costs. Dialysis and transplantation therapies are not globally accessible and the risk of recurrent glomerular disease following transplantation can be as high as 30% (4). There are currently limited therapies to slow the progression of glomerular disease and, therefore, a significant need to build our understanding about both normal glomerular function and the dysfunction associated with pathology.

The integrity of the glomerular filtration barrier depends on both cell–cell adhesion and cell–matrix adhesion, which have been predominantly investigated in podocytes, although undoubtedly critical for the glomerular endothelium. Cell–cell and cell–matrix adhesion receptors connect adjacent cells or matrix ligands to the cellular cytoskeleton, and they are vital conduits for the transmission of signals in and out of cells. These receptors are highly conserved in evolution and are a fundamental requirement for tissue development. The glomerular filtration barrier is a highly complex structure, and we require deep understanding to appreciate how the barrier is formed during development, regulated in health, and disrupted in disease. This review focuses on the importance of podocyte adhesion for a healthy glomerulus. Cell–matrix adhesion is introduced initially with a review of the glomerular extracellular matrix (ECM) and our understanding about cell adhesion to ECM ligands. Cell–cell adhesion follows with a review of the unique podocyte slit diaphragm. Finally, we discuss the prospects for therapy to target defects in adhesion for patients with glomerular disease.

## The Glomerular Extracellular Matrix

Extracellular matrix is essential for multicellular life providing a structural scaffold with appropriate mechanical properties to support adjacent cells (5). It comprises a complex network of glycosaminoglycans and fibrous proteins, which are synthesized and secreted by cells. Podocytes and glomerular endothelial cells adhere to ECM networks via cell surface receptors. This cell–ECM interface forms a signaling platform that controls all aspects of cell fate decisions, including shape, growth, differentiation, and survival (5, 6). In addition to this signaling platform, the ECM modulates cell–cell signaling by sequestering secreted growth factors and cytokines, forming reservoirs for controlled release (5).

Basement membranes are condensed sheets of ECM with a supramolecular assembly built around two major networks of laminin and collagen IV. In the glomerulus, ECM is organized as the GBM of the capillary walls and basement membrane of Bowman’s capsule, in addition to the loose mesangial ECM between capillary loops (Figure 1). The mature GBM is thicker than most basement membranes (300–350 nm in humans), and it represents a fusion of two membranes, one derived from podocytes and the second from endothelial cells during glomerular development (7). The study of human glomerular disease led to the discovery of tissue-restricted isoforms of laminin and collagen IV in the mature GBM, and these are key components of this specialized extracellular niche (8, 9).

Laminins are self-assembling heterotrimeric glycoproteins (10, 11) and an absolute requirement for basement membrane formation (12, 13). All laminin heterotrimers contain α, β, and γ chains. The trimeric protein has a cruciform shape with one long arm and three short arms and the short arms contain the amino terminal (LN) domains of the laminin heterotrimer. These short arms also form the nodes within the laminin network, exclusively via interactions between the chain specific LN domains (14–16). The long arms contain the globular (LG) domains with cell surface receptor binding sites (17–21).

The developing GBM contains laminin α5β1γ1 (laminin-511), but the mature GBM comprises predominately laminin-521. A complete laminin-521 network is the key for a functional GBM as mutations in *LAMB2*, the gene encoding the laminin β2 chain, cause Pierson syndrome in human beings. Affected individuals have a spectrum of pathology dependent on the type of mutation with truncating mutations causing congenital nephrotic syndrome, microcoria, muscular hypotonia, and neurodevelopmental deficit (22, 23). *Lamb2* mutations in mice are also associated with glomerular dysfunction. Mice with null mutations die after 3 weeks of age with severe proteinuria and neuromuscular defects (24). These animals have accumulation of ectopic laminin chains in the GBM, including α1, α2, α3, β1, β3, and γ2; however, these chains do not compensate for the loss of the β2 chain, possibly due to low expression or the absence of a complete laminin network (25). The theory that insufficient expression of laminin chains accounts for the observed lack of compensation is supported by the finding that podocyte overexpression of *Lamb1* in *Lamb2* null mice ameliorates proteinuria (26).

Unlike the laminin network, the collagen IV network is dispensable for basement membrane formation; however, it appears to be important for strength and stability (27). Collagen IV forms heterotrimers comprising three alpha chain combinations (α1α1α2, α3α4α5, or α5α5α6). Each alpha chain contains three distinct domains; an amino terminal 7S domain rich in cysteines and lysines, which is essential for inter-chain crosslinking through disulfide bonds and lysine/hydroxylysine crosslinks; a long collagenous repeat domain, around 1400 amino acids in length; and a carboxy terminal non-collagenous domain (NC1) (28). A novel chemical bond, not previously identified in biomolecules, the sulfilimine bond (-S =N-), was recently discovered in collagen IV. This bond crosslinks lysine/hydroxylysine-211 and methionine-93 of adjoining protomers in the NC1 domains of both collagen IV α1α1α2 and α3α4α5, which may provide additional resistance of the network to mechanical strain (29). Furthermore, peroxidasin, an enzyme found in basement membranes, catalyzes the formation of the sulfilime bond (30), and in ground breaking recent work, ionic bromide was shown to be a cofactor required for peroxidasin-catalyzed formation of the sulfilimine crosslinks in collagen IV networks (31), thus describing the first known essential function for bromine in animals.

From the capillary loop stage of glomerular development, the GBM comprises predominantly α3α4α5 networks of collagen IV, and as with laminin, the developmental collagen IV transition is critical for GBM maturation. Mutations leading to a reduction or absence of the α3α4α5 networks cause human Alport syndrome characterized by a renal phenotype of hematuria, proteinuria, and progressive renal failure (28, 32). The GBM in Alport syndrome has increased collagen IV α1α1α2, which is unable to compensate for the lack of the α3α4α5 network. As a consequence, the GBM develops splits and a typical basket-weave appearance, leading to speculation that mechanical strain cannot be tolerated perhaps due to fewer disulfide bonds in the α1α1α2 network relative to α3α4α5 and consequently a weaker GBM. This concept is further supported by the observation that reducing mechanical strain in the glomerulus with angiotensin-converting enzyme (ACE) inhibitors, which lower blood pressure as well as transcapillary filtration pressure, significantly delays disease progression in Alport syndrome (33–35).

The laminin and collagen IV networks are indirectly linked via nidogens (36) and the heparan sulfate proteoglycans, perlecan (37, 38), and agrin (39). Podocyte-specific deletion of agrin from the GBM resulted in a significant reduction in the negative charge associated with the barrier, however, alone or combined with knockout of perlecan, agrin deletion was not associated with proteinuria, therefore questioning the role of charge selection in glomerular filtration (40). Nidogen 1 and 2 are dumbbell-shaped proteins and bind to both laminin and collagen IV. Mice with knockout of either nidogen 1 or 2 are viable and have normal basement membranes. Deletion of both isoforms, however, causes perinatal lethality (41). This is consistent with a degree of redundancy in their ability to bind collagen IV and laminin. Surprisingly, the GBM has a normal appearance even in the double (*Nid-1*, *Nid-2*) knockout. This suggests that nidogen is dispensable for the formation of the GBM, but again it may be required for the GBM to resist mechanical strain. Taken together, it is likely that agrin, perlecan, and nidogens are important for overall basement membrane strength by contributing to the crosslinking of the collagen and laminin networks to each other and to the cell surface.

While these and other investigations detail the composition of the glomerular ECM, it has been more challenging to elucidate the relative position of ECM proteins in the GBM. However, a systematic analysis of the spatial arrangement of ECM components within basement membranes, with respect to each other and to their cell–adhesion receptors, was recently performed using super resolution microscopy (42). This investigation found two separate laminin networks, one produced by podocytes the other produced by endothelial cells. The collagen IV α3α4α5 network was distributed along the center of the GBM alongside nidogen, consistent with its putative crosslinking function. The human GBM is approximately twofold thicker than the mouse GBM, and interestingly, this study found increased thickness of the human collagen IV a3a4a5 network, and potentially, an additional layer of laminin-521 closer to the center of the GBM (42).

Thus, candidate-based investigations of the glomerular ECM have significantly advanced our understanding about key components. However, more recently unbiased, global approaches have shown that the glomerular ECM is a highly complex extracellular niche. In our own proteomic analysis of human glomerular ECM, we identified 144 structural and regulatory ECM proteins and found that more than 50% were expressed in the GBM (43). Together with the analysis of cell-derived ECM produced by glomerular cells in culture, we found a common core of highly connected and clustered ECM proteins, which may be important for ECM assembly (44). Overall, the glomerular ECM is a complex scaffold of interacting proteins, which are likely to be highly dynamic and are unique in order to support the complex function of the glomerular filtration barrier.

## Podocyte Adhesion to the GBM

**Figure 3:**
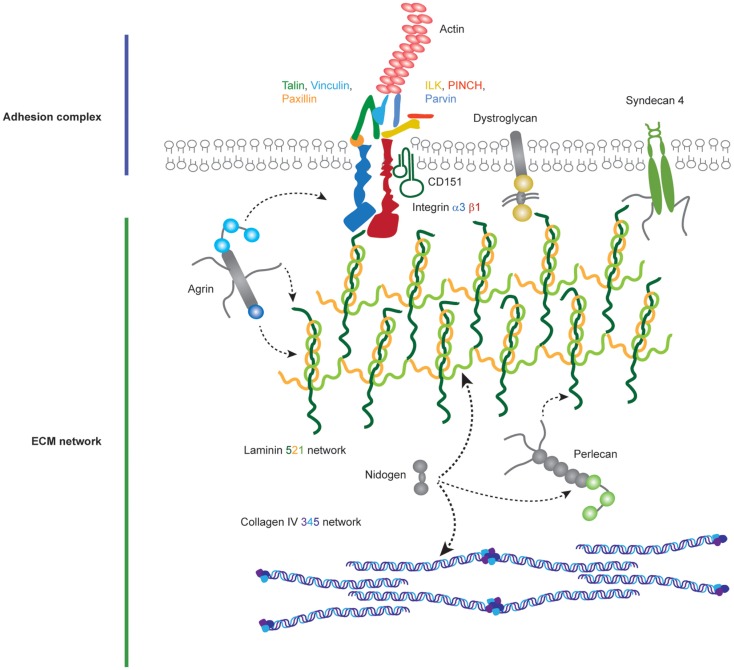
**Molecular components of the podocyte cell–matirx interface**. Podocytes adhere to the underlying GBM using transmembrane adhesion receptors. The laminin-binding integrin α3β1 and the associated tetraspannin CD151 are highly expressed on the podocyte cell surface, in addition to adhesion receptors for other ECM ligands. Adhesion complexes form when activated integrins recruit adaptor, scaffold, and signaling proteins to their cytoplasmic tails. Integrins link to the actin cytoskeleton via two major axes; talin, vinculin, paxillin and integrin-linked kinase (ILK), PINCH, parvin. Podocytes attach to an ECM network containing laminin-521 networks, collagen IV α3α4α5 networks, agrin, perlecan, and nidogen.

In order to adhere to the GBM, podocytes and endothelial cells utilize transmembrane adhesion receptors and the cell–matrix adhesion of podocytes was recently reviewed elsewhere (45). Adhesion receptors contain extracellular domains, which can bind to specific ECM proteins and intracellular domains that recruit effector proteins and link adhesion receptors to the cell cytoskeleton (Figure 3). A major family of proteins responsible for cell–ECM adhesion is the integrins.

Integrins are αβ-heterodimers that propagate signals from within the cell to the immediate extracellular environment in addition to outside–in signaling. All integrins link to the actin cytoskeleton, with the exception of α6β4, which links to intermediate filaments (6). Conformational changes in these receptors are central to the regulation of integrin receptor activity. Integrins adopt either a low affinity bent conformation, a primed or active high affinity extended conformation, or a ligand occupied state (46). Integrins form 24 different αβ combinations (6), which have differing affinities for ECM ligands and also have differential recruitment of proteins to their cytoplasmic domains (47). Laminin-binding integrins include α1β1, α2β1, α3β1, α6β1, α10β1, and α7β1, and the collagen binding integrins are α1β1, α2β1, α10β1, α11β1, and αXβ2 (48).

Upon integrin engagement of the ECM, there is integrin clustering and activation. Integrins lack intrinsic enzymatic activity; therefore, in order to propagate signals into the cell, active integrins must recruit a number of adaptor and effector proteins into sites known as focal adhesions. At least 232 protein components are recruited to adhesion complexes in a cell type and context dependent manner, demonstrating the potential for adhesion signaling to bring about different cellular outcomes (49). In addition, global analyses of adhesion complexes using mass spectrometry suggest an even larger number of proteins may be recruited to focal adhesions (50–52). Examples of the groups of proteins recruited to sites of active integrins are adaptors, actin remodeling proteins, signaling proteins, GTPase regulators including guanine-nucleotide exchange factors (GEFs) and GTPase activating proteins (GAPs) in addition to numerous serine, threonine and tyrosine kinases, and phosphatases. This signaling nexus controls all aspects of cell fate and so does not just act as a mere anchoring point for cell attachment but also a signaling hub to alter cell behavior. Furthermore, integrin association and cross talk with other transmembrane receptors such as syndecans increases the potential for regulation of integrin-mediated adhesion (53, 54).

The α3β1 heterodimer is the most highly expressed integrin on the podocyte cell surface, and is thought to be the most important link between the podocyte and the GBM (55–57). Homozygous mutations in *ITGA3*, the gene encoding integrin α3, in humans leads to congenital nephrotic syndrome, interstitial lung disease and epidermolysis bullosa (58) with defects in the GBM. In addition, a mutation in *ITGA3* causing a gain of glycosylation and preventing α3β1 dimer formation causes fatal interstitial lung disease and congenital nephrotic syndrome (59). This phenotype is recapitulated in mice lacking the integrin α3 subunit, which die within the first day of life due to developmental defects in the kidneys and lungs, including loss of specialized podocyte morphology and thickened irregular GBMs (60). Moreover, podocyte-specific deletion of *Itga3* in the mouse also resulted in a disorganized GBM with thickening and protrusions and an inability of podocytes to form mature foot processes (61). Human mutations affecting integrin-β1 have not been described, to date, and this may be due to embryonic lethality since integrin-β1 forms at least 12 heterodimers. However, the role of this integrin has been studied with podocyte-specific deletion of *Itgb1* in the mouse. This resulted in a severe phenotype of proteinuria from birth and renal failure by 3 weeks featuring both glomerular and tubular pathologies (62, 63).

The tetraspannin CD151 binds tightly to integrin α3β1 (64) and humans with mutations in *CD151* develop hematuria and proteinuria progressing to end-stage kidney disease in addition to pretibial epidermolysis bullosa, sensorineural deafness, and β-thalassemia minor (65). In mice, deletion of *Cd151*, both globally and specifically in podocytes, caused early proteinuria with abnormalities of the GBM loss of podocyte foot processes, glomerulosclerosis, loss of podocytes, and renal failure. This phenotype, however, is dependent on the genetic background of the mice, with *Cd151*-knockout mice on the FVB background displaying the pathological phenotype (61, 66, 67). It is hypothesized that CD151 increases the strength of podocyte adhesion to the GBM via integrin α3β1 engagement with laminin-521. *Cd151*-knockout mice on the C57BL/6 background do not spontaneously develop renal failure but when challenged with induced hypertension, they develop significant proteinuria. Furthermore, treatment of the susceptible *Cd151*-knockout FVB strain with ACE inhibitors ameliorated progression of renal failure. In addition to *in vivo* experiments, *in vitro* experiments showed that podocytes lacking CD151 lose their resistance to shear stress when cultured on laminin (67). This evidence supports a crucial role for integrin α3β1 as a major adhesion receptor, and in combination with CD151, a complex necessary to withstand mechanical forces within the glomerulus.

The laminin-binding integrin α6β4 may also be the key for the development and maintenance of the glomerular filtration barrier. Human mutations in *ITGB4* have been described and associated with junctional epidermolysis bullosa and pyloric atresia (68, 69). In one of these patients, there was coincident nephrotic range proteinuria and the study demonstrated reduced expression of integrin-β4 in podocytes; however, the possibility of an alternative genetic explanation for the glomerular dysfunction in this case remains possible. Integrin αvβ3 has also been implicated in glomerular dysfunction (70, 71). This fibronectin receptor was shown to be activated following the induction of urokinase receptor (uPAR) signaling leading to increased podocyte motility and activation of GTPases. In a subsequent study, the same team identified soluble uPAR as a potential pathogenic mediator of disease in nephrotic syndrome associated with focal segmental glomerulosclerosis (FSGS) where there was also activation of integrin-β3 (72). These studies raise the possibility that abnormal integrin activation in the podocyte alters cell motility and this signaling pathway could potentially be targeted therapeutically.

In addition to the integrin family of adhesion receptors, transmembrane heparan sulfate proteoglycan receptors, such as the Syndecan family, are key regulators of cell-ECM interactions (54). Cooperation of integrins and syndecans in adhesion formation has been shown on a variety of ECM ligands including fibronectin, vitronectin, and laminin (73–76). Syndecans regulate intergrin trafficking to the cell surface (77), a process used by cells to regulate adhesion formation and disassembly (78–80). In addition to modulating integrin dynamics, syndecans facilitate growth factor binding to their receptors (81, 82). In podocytes null for EXT1, a key molecule in herparan sulfate glycosaminoglycan assembly, adhesion complexes were reduced in size, the actin cytoskeleton was rearranged, and cell surface syndecan 4 upregulated (83). However, mice null for EXT1 specifically in podocytes do not develop significant proteinuria, despite some podocyte abnormalities, including a degree of foot process effacement (84). In podocytes, autocrine signaling by the soluble vascular endothelial growth factor receptor, sFLT1, also causes actin rearrangements and this is associated with phosphorylation of both syndecan 1 and 4 within their EFYA motifs (85). Thus, there is accumulating evidence that syndecans contribute to cell–matrix adhesion and signaling in podocytes.

Dystroglycan is a cell surface adhesion receptor and comprises a highly glycosylated extracellular α-dystroglycan subunit, which can bind to laminins, and a non-covalently linked intracellular β-dystroglygan subunit that links to the actin cytoskeleton via an interaction with utrophin (86, 87). Dystroglycan is expressed by podocytes (88) and the expression pattern is altered in glomerular pathologies (89, 90). Therefore, it seemed likely that dystroglycan was important for podocyte adhesion; however, defective glycosylation of α-dystroglycan, which abrogates α-dystroglycan-laminin interactions does not cause proteinuria, only mild podocyte foot process effacement (91). Furthermore, podocyte-specific deletion of dystroglycan in mice caused only mild GBM thickening (92). These data suggest that dystroglycan is not a critical adhesion receptor in podocytes.

## Focal Adhesion Complexes

A number of proteins link integrins to the actin cytoskeleton and form focal adhesions (Figure 3). One such is talin-1, a 270 kDa protein comprising an N-terminal globular head and flexible rod domain. The head domain contains a FERM domain with binding sites for the integrin-β subunit cytoplasmic tail, F-actin, focal adhesion kinase (FAK), and PIPK1γ90. The rod domain contains an additional binding site for integrin, actin binding sites, and multiple vinculin binding sites, and this domain can also bind to RIAM (93). Finally, the C-terminal domain contains helices responsible for talin dimerization. Binding of talin to the cytoplasmic tail of β-integrins triggers a conformational change in the extracellular domain of integrins, which amplifies the affinity of the integrin for the ECM. Talin dependent recruitment of further proteins to active integrins causes the consequent formation of focal adhesions (94). Talin-1 expression in podocytes is required for the specialized actin morphology of foot processes. Podocyte-specific *Tln1*-knockout mice develop proteinuria and die within 10 weeks. These mice, however, did not have major defects in integrin β1 activation or podocyte adhesion. Nevertheless, the actin cytoskeleton was perturbed, and there was podocyte foot process effacement. These data show that talin-1, a protein known to be important in adhesion formation and linkage to the actin cytoskeleton *in vitro*, is a key player in relaying signals from integrins to the actin cytoskeleton in podocytes *in vivo* (95).

Another key adaptor protein involved in integrin-mediated adhesion complex formation is vinculin, a 123 kDa protein recruited by talin to focal adhesions and capable of binding to the actin cytoskeleton (96). Vinculin comprises an N-terminal head, proline rich neck, and a C-terminal tail domain (97, 98). Cytoplasmic vinculin assumes an autoinhibited inactive conformation (99) and following talin recruitment, vinculin undergoes a conformational change revealing an open active state (100). Vinculin is a force regulator and when extended by forces applied through actin, there is subsequent recruitment and release of focal adhesion proteins (101). This conformational change allows vinculin to directly interact with a number of proteins including α-actinin, Arp2/3, actin, and paxillin (102, 103). The vinculin head domain modulates integrin clustering, whereas the tail domain links to actin. Considering vinculin is an important link between integrins and the actin machinery, this protein may have a key role in the force sensing by podocytes via integrin α3β1.

Paxillin is another component of focal adhesions and it acts as a scaffolding protein. It contains multiple protein-binding modules, many of which are regulated by phosphorylation. It localizes to focal adhesions through phosphorylation of its C-terminal LIM domains (104, 105). Paxillin is an important molecular adaptor; its N-terminus controls most of its signaling activity that provides docking sites for vinculin, FAK, Src, and Crk. Paxillin is recruited to focal adhesion by talin (106) and brings about spatiotemporal control of Rho family small GTPases by recruiting numerous GEFs and GAPs (107).

Another highly studied focal adhesion protein is FAK. FAK is non-receptor tyrosine kinase, recruited to focal adhesions by talin and paxillin (108). FAK has a number of roles at focal adhesion sites, including recruitment of p130Cas, Crk1/2, and Src family kinases (109). Global deletion of FAK in mice is lethal in embryogenesis, causing a profound migration defect (110). The importance of FAK in podocytes was highlighted by the observation that FAK is phosphorylated upon podocyte injury (111). Surprisingly, podocyte-specific deletion of FAK in mice leads to a normal phenotype; however, these mice are protected from proteinuria and podocyte injury after experimental podocyte insults (111). Additionally, podocyte injury was reduced when a FAK inhibitor was administered in a mouse model of glomerular injury (111). A role for FAK has also been found in Alport syndrome where ectopic laminins, α1 and α2, accumulate in the GBM. Laminin α2 caused phosphorylation of FAK at Y397, and this phosphorylation was associated with upregulation of the proteases MMP 9 and 10 and GBM defects (112). FAK inhibition reduced proteinuria, MMP levels, and GBM defects (112). These data, therefore, support a role for FAK in glomerular dysfunction.

Integrin-linked kinase binds directly to the integrin β1 cytoplasmic tail and is important for signal transduction at adhesion sites (113). ILK was originally identified as a kinase, but increasing evidence suggests that is a pseudokinase (114–119). In fact, the C-terminal kinase homology domain of ILK mediates multiple protein–protein interactions at adhesion sites, including interactions with α/β/γ-Parvin (120, 121). ILK also contains five ankyrin domains that mediate interactions with PINCH-1/2 (122–124). Kindlin 2 is another ILK interacting protein, which is expressed in podocytes, localizes to focal adhesions and through either ILK/PINCH/parvin or migfilin–filaminin interactions binds to the actin cytoskeleton (125–127). It is through this scaffolding role that ILK orchestrates focal adhesion signaling. The ILK/PINCH/parvin complex influences the actin cytoskeleton (128), in addition to negatively regulating cell contractility (129). Total loss of ILK or PINCH in mice is lethal in embryogenesis, due to failure in epiblast polarization (128, 130). The interaction between ILK and α-parvin is required for kidney development. Mutations in ILK K220 disrupt α-parvin binding and cause renal agenesis (131). Furthermore, a similar phenotype is observed when α-parvin is genetically deleted in mice (131). Podocyte-specific loss of ILK in mice causes GBM defects, loss of slit diaphragms, and podocyte foot process effacement (132, 133). Moreover, ILK interacts with nephrin at podocyte cell junctions, suggesting that ILK is a potential link between cell-cell and cell-ECM adhesion signaling (132). Finally, increased expression of ILK is observed in a variety of glomerular diseases (134, 135). This evidence strongly supports an important role for ILK in adhesion signaling in podocytes.

**Figure 4:**
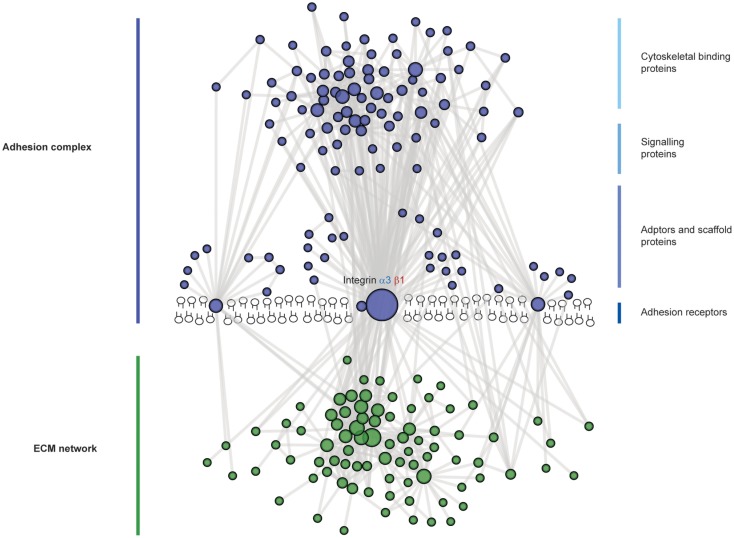
**The complexity of adhesion signaling, a protein–protein interaction view**. A predicted protein–protein interaction network of adhesion signaling complexes in podocytes. Nodes represent proteins and lines between nodes represent experimentally derived protein–protein interactions. Extracellular matrix (ECM) proteins are green in color and adhesion signaling proteins are blue in color. Integrins do not have intrinsic enzymatic activity; however, through conformational changes, integrins can recruit tens to hundreds of different proteins to adhesion sites. These adhesion sites are hubs for cellular signaling, controlling all aspects of cell fate. Adhesion signaling is determined by the composition and physical properties of the extracellular matrix.

Overall cell adhesion to the GBM occurs at a complex cell–matrix interface (Figure 4). A wide range of scaffolding proteins localize to focal adhesions and transmit information regarding the extracellular environment via recruitment of effectors including kinases, and GTPases. The cellular adhesome has been predominantly investigated in the context non-adherent cells or fibroblasts but less so for epithelial cells, and similar analyses in glomerular cells will help to build our understanding about the key cellular components that are involved in cell–matrix adhesion in the glomerulus. These studies have the capacity to identify unexpected and novel proteins at adhesion sites, which are considerably more complex than previously thought.

## Adhesion at the Podocyte Slit Diaphragm

The junction between adjacent podocyte foot processes is termed the slit diaphragm and it is visible by electron microscopy as an electron dense structure close to the GBM. This specialized junction is thought to connect the entire length of adjacent foot processes providing a structural component to the filtration barrier. From early ultrastructural studies, a zipper-like substructure was described where protein bridges emanating from the podocyte plasma membrane link to a central filament in a lattice arrangement with rectangular pores (136). The calculated cross-sectional dimensions of these pores was 4 × 14 nm, approximately the size of an albumin molecule, and therefore, consistent with the observations from tracer studies using ferritin and dextrans that the slit diaphragm contributed significantly to the retention of macromolecules within the circulation (137, 138). Following these seminal ultrastructural and tracer studies was the discovery of the first unique slit diaphragm protein nephrin by positional cloning of the gene in congenital nephrotic syndrome of the Finnish type and leading, to further refinement of the zipper-like model of the podocyte cell junction (139, 140). More recent ultrastructural studies have used scanning electron microscopy to describe circular and ellipsoidal pores in the central region of the slit diaphragm, with a mean diameter of 12.1 nm (141). Interestingly, the same study demonstrated an increase in the size of some of these pores with proteinuria, perhaps providing an explanation for the increased transit of macromolecules across a defective filtration barrier.

Many studies have also shown that the architecture of the podocyte changes dramatically in human glomerular disease with flattening of the actin-rich foot processes and loss of slit diaphragms. Similar changes are observed in animal studies of puromycin aminonucleoside (PAN)-induced nephrotic syndrome or nephrosis (142). While these dramatic morphological changes are associated with a profound barrier defect, remarkably these changes seem to completely reverse especially in the subset of patients with nephrotic syndrome who respond to treatment with glucocorticoids.

## Components of the Podocyte Slit Diaphragm

The first junctions to form in podocytes are apical and have been described as tight junctions (143). During glomerular development, the junctional complexes descend toward the GBM and widen to become the mature slit diaphragm. These are highly specialized and unique junctions and many studies have identified components associated with more classical types of cell junctions. The zona occludens protein (ZO-1) was one of the first proteins found to localize to podocyte foot processes using immunogold labeling and electron microscopy (144). Using immunostaining of murine podocytes in culture and rat glomeruli, podocyte cell junctions were also shown to contain classical components of adherens junctions including cadherin-3 and α-, β-, and γ-catenins (145). The tight junction components JAM-A, occludin, and cingulin were also found to be associated with slit diaphragms (146) and the same study reported that PAN nephrosis increased the expression of these tight junction components. There is also a report of the gap junction protein connexin-43 localizing to the podocyte slit diaphragm, and it was found to be upregulated in the early phase of PAN nephrosis (147). Taken together, these findings suggest, not surprisingly, that there is context-dependent composition of these junctions.

**Figure 5:**
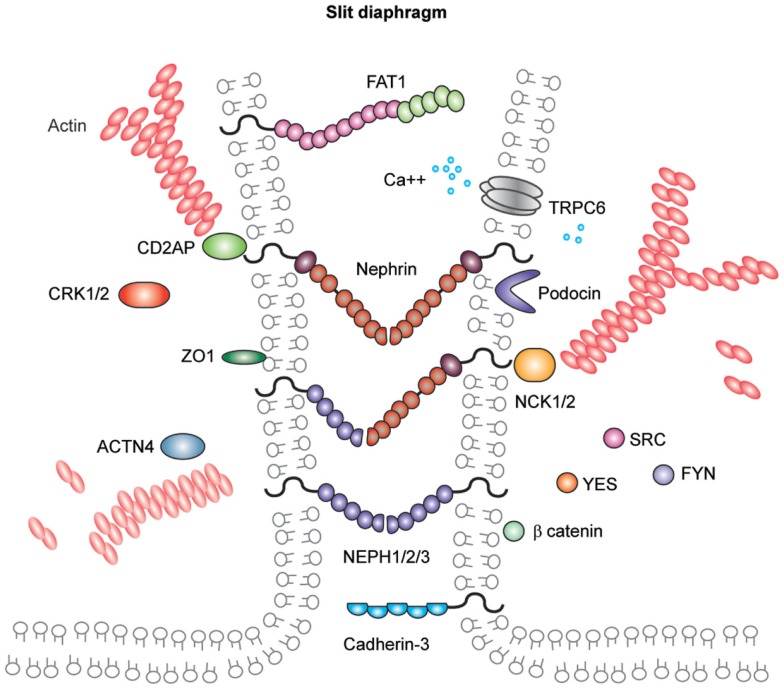
**Molecular components of the podocyte slit diaphragm**. Podocyte slit diaphragms contain unique components, including nephrin, Neph family proteins, and podocin in addition to components of tight, gap, and adherens junctions, such as the cadherin-3 and FAT1. The adaptor proteins Nck1/2, CD2AP, and Crk1/2, Src family kinases, in addition to many more kinases and actin binding proteins localize to the podocyte slit diaphragm. The interactions between nephrin and other Neph proteins are thought to be homo- and heterophilic and recent structural analysis of the orthologs SYG1/2 has suggested an angled conformation (152) as depicted in this schematic.

In addition to components associated with other cell junctions, the slit diaphragm also contains unique proteins (Figure 5). Nephrin and the homologs Neph-1, Neph-2, and Neph-3 are known members of this cell junction and are comprehensively reviewed elsewhere (148). They are members of the immunoglobulin superfamily of cell-adhesion receptors and are involved in the development of specialized junctions in neurons and at the slit diaphragm (148). Orthologs of these proteins are expressed in *Drosophila* nephrocytes (149), which have nephrocyte diaphragms structures with very similar composition to the mammalian slit diaphragm (150). Interestingly, birds lack nephrin and Neph-3 but do form slit diaphragm-like structures (151). Further back in evolution, *Caenorhabditis elegans* expresses the orthologs SYG-1 (Neph1) and SYG-2 (nephrin), and these are required for synapse formation and specificity. Investigation of the crystal structures of these orthologs revealed SYG-1 homodimers with a conserved binding interface and an unusual, angled geometry in the heterophillic SYG1/2 complex (152). The crystal structures of nephrin and Neph homologs remain unresolved; however, there is some evidence for homophilic nephrin interactions. These interactions were detected using recombinant nephrin protein and surface plasmon resonance, and they were increased in the presence of calcium (153).

Nephrin and Neph-1 are requisite components of the slit diaphragm. Mutations in nephrin cause human congenital nephrotic syndrome (139), which is most common in Finland, although many mutations have now been described in individuals with later onset of disease and from a diverse ethnic background (154). Infants require albumin infusions to maintain intravascular volume and ultimately proceed to removal of their kidneys prior to dialysis and transplantation. This disease phenotype is mimicked in the mouse where deletion of *Nphs1* leads to early massive proteinuria and the mice die within 24 h. Ultrastructural analysis of their glomeruli has revealed the absence of slit diaphragms and foot process effacement (155). *Neph1* deletion in mice is also associated with perinatal lethality with proteinuria and podocyte foot process effacement (156). As yet, no human mutations in *NEPH1* have been described but it is more widely expressed than nephrin, and therefore, mutations may be incompatible with life.

Other notable members of the slit diaphragm complex include podocin, a stomatin family protein that is also mutated in patients with early onset nephrotic syndrome (157). Podocin is important for the recruitment of proteins to the slit diaphragm complex and for facilitating signaling (158). CD2AP is an adaptor protein and its role in maintaining the integrity of the filtration barrier was first described in mice (159) and more recently in human disease (160). CD2AP and CIN85 appear to be important for the balance of receptor tyrosine kinase signaling in podocytes (161). FAT atypical cadherin-1 has also been shown to regulate barrier formation and mice lacking this component have significant glomerular defects, in addition to eye and brain abnormalities (144).

**Figure 6:**
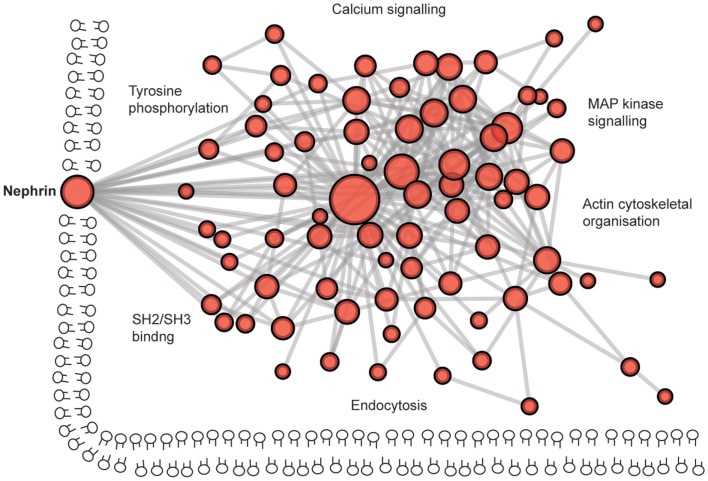
**The complexity of the podocyte slit diaphragm – a network view**. A predicted protein–protein interaction network based on interactions of proteins localized to the slit diaphragm. Nodes represent proteins and lines between nodes represent experimentally derived protein–protein interactions. Labels represent Gene Ontology vocabularies significantly (*P* < 0.05) enriched in proteins localized to the slit diaphragm.

The protein complex at the slit diaphragm includes the components that make the connections between adjacent podocyte foot processes in addition to the more dynamic network of proteins that assemble intracellularly (Figure 6). To identify novel components of this complex, unbiased approaches with mass spectrometry have led to the discovery of proteins including IQGAP (162). These global analyses will continue to assist in the identification of more unexpected components of these junctions as methods to isolate and analyze the junctions improve. To give an indication of the scale of the components, a recent bioinformatic analysis of the cadhesome has predicted an assembly of 170 components many of which may be cell type and context specific (163).

## Signal Transduction at the Slit Diaphragm

Over the past 16 years since the discovery of nephrin, a growing number of proteins have been linked to signaling at the slit diaphragm and there are several recent, comprehensive reviews (164, 165). Phosphorylation of nephrin and Neph family proteins is the key to signal transduction. Tyrosine phosphorylation by Src family kinases initiates a signal cascade and indeed the deletion of Fyn resulted in barrier dysfunction (166). Phosphorylation by Fyn leads to the recruitment of a number of proteins including the adaptor proteins Nck1/2 (167, 168), Crk1/2 (169), CrkL (170), and Grb2 (171, 172) in addition to PI3-kinase (173, 174). Following recruitment, Nck binds phosphorylated nephrin and leads to actin reorganization via the actin nucleation factor N-WASP (175). The receptor Robo2 also links to Nck and is expressed in podocytes (176). This receptor was found to inhibit actin reorganization and it appears to negatively regulate signaling via nephrin and Nck therefore to reduce podocyte foot process effacement. Crk is recruited to phosphorylated nephrin via p130Cas and deletion of Crk1/2 attenuated podocyte foot process effacement in a glomerular injury model (169). The p85 regulatory subunit of P13-kinase also interacts with nephrin leading to downstream activation of Akt (173) and subsequently to actin reorganization (174). Demonstrating the importance of signaling via Akt, deletion of the Akt2 isoform was associated with barrier dysfunction (177) and Akt phosphorylation in podocytes follows insulin signaling (178) and is also linked to mTOR signaling (177).

Regulation of the podocyte actin cytoskeleton is, therefore, the key to maintaining barrier integrity and actin reorganization in podocytes is likely to relate directly to the dramatic podocyte foot process effacement, which is seen across the spectrum of disease. The actin crosslinking protein alpha-actinin-4 has been associated with human nephrotic syndrome and mutations in *ACTN4* are associated with adult onset FSGS (179). Here, the mutated alpha-actinin-4 protein binds filamentous actin more strongly than the wild type protein, indicating that actin regulation is important for normal podocyte function. Another class of actin regulators is the GTPases, which in turn are regulated by GEFs, GAPs, and GDIs. Podocyte-specific deletion of the GTPase RhoA did not result in a barrier defect (180); however, activation of RhoA has been described in a number of glomerular injury models in addition to human disease including mutations in the formin INF2, leading to the commonest cause of adult onset FSGS (181). A number of studies have now investigated the role of Rac1 in podocytes, which is required for lamellipodia formation. Rac1 is not essential for glomerular development but overexpression leads to barrier dysfunction either with constitutive activation of Rac1 (182) or RhoGDI-alpha knockout (183). However, a number of studies have also shown that Rac1 is protective (170, 184) and it is likely that a fine balance is required. The GTPase CDC42 is linked to the formation of filopodia and its absence results in early barrier dysfunction (180, 185) and this may be due to links with apical-basal polarity proteins, which are also required for slit diaphragm formation (186).

The dynamic regulation of this specialized cell junction undoubtedly requires quality control and recycling of components, and it was recently shown using rat glomeruli that the turnover rates of slit diaphragm proteins was high and was regulated by atypical protein kinase C (aPKC) (187). Accordingly, the endocytic pathway components dynamin, synaptojanin, and endophilin have been shown to be important for maintaining barrier function (188). The correct localization of proteins is also the key, and nephrin localization at the plasma membrane requires the endocytic protein Myo1c (189). The long-tailed myosin, Myo1E, may also contribute to endocytosis in podocytes (190, 191). The role of calcium signaling at the slit diaphragm is also important and mutations in the transient receptor potential cation channel *TRPC6* have been associated with adult onset FSGS (192, 193).

There are still many research questions to address in order to understand how the specialized slit diaphragm is formed during development, regulated in health, and disrupted in disease. Chemical and mechanical factors in the microenvironment are likely to be the key, and although the barrier regulation by growth factors has not been discussed here, there is growing evidence for the important roles of vascular endothelial growth factor (VEGF-A) (85) and insulin (178). Regarding mechanical cues, it would seem likely that filtration forces contribute to the regulation of the barrier. The molecules spanning the slit diaphragm are directly exposed to force and it would be intriguing to determine whether some of these components respond to force in a similar manner to VE cadherin, which was recently investigated using a tension biosensor and shown to stretch in endothelial cells exposed to sheer stress (194). Understanding some of these basic mechanisms of regulation will be required to identify specific therapeutic strategies to maintain or restore glomerular function.

## Prospects for Adhesion-Based Therapy for Glomerular Disease

Cell adhesion is clearly important to maintain normal barrier function but what are the prospects for adhesion-based therapies? Current therapies for glomerular disease include both immunomodulation and inhibition of the renin-angiotensin-aldosterone (RAAS) signaling pathway. RAAS pathway inhibition with ACE inhibitors and angiotensin receptor blockers (ARBs) are thought to act primarily by reducing glomerular hydrostatic pressure. Immunomodulatory drugs such as glucocorticoids and calcineurin inhibitors were initially thought to act via immune cells; however, these agents may also directly target the podocyte (195). The effects of existing and efficacious therapies on podocyte adhesion have not been formally tested, although there are some intriguing observations. Spironolactone (an inhibitor of aldosterone) was shown to reduce the urinary excretion of podocytes in a rat model of diabetic nephropathy (196), and this presumed reduction in podocyte detachment was associated with upregulation of integrin α3. Furthermore, in a study of human podocytes in culture, the glucocorticoid dexamethasone was shown to increase nephrin expression (197).

The manipulation of integrin activation as a possible route to therapy has been suggested in a series of recent studies. Activation of integrin-β3 has been implicated in the pathogenesis of nephrotic syndrome associated with FSGS. In a subset of patients with FSGS, there is strong evidence for the role of a circulating and disease-causing factor, which can lead to recurrence of primary disease in transplanted kidneys. Soluble urokinase receptor (suPAR) was identified as a pathogenic factor leading to activation of integrin-β3 in mouse models and human disease (72) leading to the suggestion that therapy for this condition could involve inhibition of the suPAR-integrin-β3 interaction by the use of small molecule inhibitors. Indeed, a beneficial effect of such an inhibitor has been shown in experimental glomerulonephritis (71).

Inactivation of integrin-β1 subunit has also been proposed, a mechanism of disease in patients with FSGS (198). Five patients with FSGS and positive B7-1 (CD80) immunostaining in glomeruli were treated with the B7-1 inhibitor Abatacept. This treatment was associated with a significant reduction in proteinuria in patients, otherwise resistant to standard therapies. While these discoveries suggest a role for manipulating signaling by adhesion receptors, given the myriad roles of these receptors, the challenge will be to target the treatment appropriately (199).

Disease-associated changes in ECM are likely to trigger a number of intracellular signaling events to influence cell adhesion. By understanding more about the pathways involved, it may be possible to target these therapeutically. In Alport syndrome, the primary molecular defect in the ECM is absence of the collagen IV–α3α4α5 network. Therefore, delivery of the wild type gene could be a potential future therapy and proof of principle was recently shown by the induction *Col4a3* in *Col4 a3*−/− mice (200). Induced expression of collagen IV-α3 slowed disease progression and improved survival providing significant optimism for the prospects of gene therapy; however, the major issue will be gene delivery to the podocyte. Abnormal outside–in signaling from the ECM could also be targeted, and in another study of Alport syndrome, inhibition of FAK in mice led to partial restoration of the GBM and a reduction in proteinuria (112).

Manipulating signaling from slit diaphragm adhesion receptors may also be a future therapeutic strategy. In patients with *NPHS1* mutations, nephrin is thought to accumulate in the endoplasmic reticulum and the hypothesis that chemical chaperones would export nephrin to the plasma membrane was tested with sodium-4 phenylbutyrate in HEK293 cells. Several mutant nephrin proteins were rescued using this strategy, and there was evidence to suggest that the mutant proteins were functional (201). More recently, inhibition of deleterious Neph-1 signaling was demonstrated with a protein transduction approach involving the introduction of the Neph-1 cytoplasmic tail, which attenuated the mislocalization of Neph-1 in two models of podocyte injury (202).

Overall, these are a selection of many encouraging observations, which indicate that adhesion-based therapy may be a prospect for glomerular disease, although there is likely to be a long journey for some of these therapies to achieve ultimate patient benefit.

## Summary

**Figure 7:**
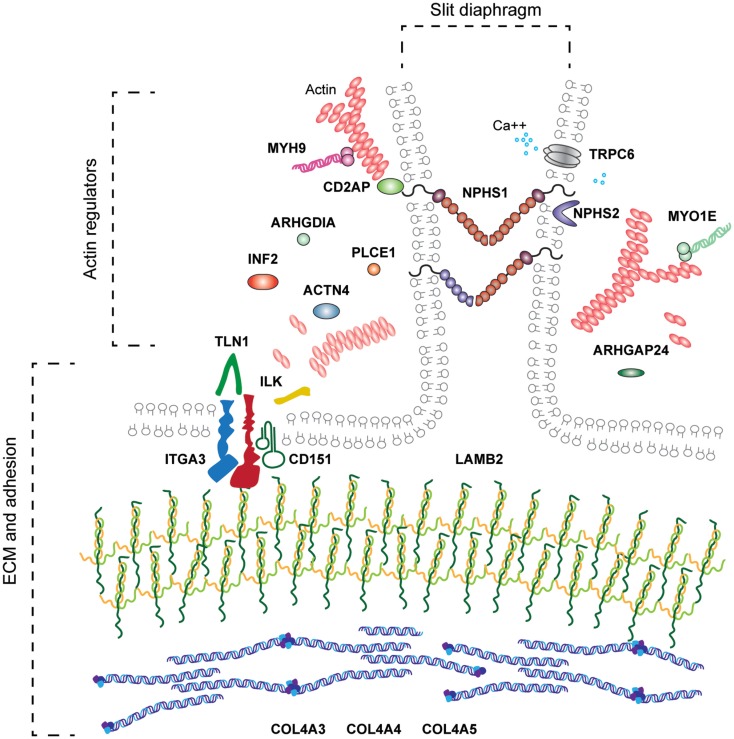
**The range of molecular functions associated with human podocytopathy**. Genetic investigations in families with nephrotic syndrome have led to the discovery of molecules required for the development and or maintenance of the glomerular filter. These include molecules associated with basement membranes, ECM adhesion, cytoskeleton, slit diaphragm, mitochondrial function, and transcription factors. This review has focused on the molecules indicated in bold.

Podocyte adhesion is evidently important for glomerular barrier integrity. Similarly, glomerular endothelial adhesion will be the key but has not yet been investigated in detail. The genetic investigations of human nephrotic syndrome have already demonstrated the range of molecular function associated with podocytopathies and severe glomerular barrier dysfunction (Figure 7) and these include defects in adhesion. Networks of signaling proteins at the cell–matrix interface and the cell–cell junctions are required to maintain barrier function, and cross talk between these adhesion complexes is likely to occur in response to mechanical and chemical signals from within the glomerular microenvironment. Improved understanding about cell adhesion in the glomerulus may lead to the identification of therapies to prevent or repair injury to this highly sophisticated filter.

## Key Points 1: Matrix

- Extracellular matrix controls cell fate decisions and growth factor signaling.
- The GBM is the ECM compartment of the glomerular filtration barrier.
- The GBM contains at least 73 components including laminin 521 and collagen IV α3α4α5.
- Laminins are essential for basement membrane assembly.
- Collagen IV is required for structural strength of basement membranes.
- The GBM contains two laminin networks separated by a collagen IV α3α4α5 network along the centre of the GBM.
- Mutations in *LAMB2* cause Pierson syndrome in humans, and affected individuals have severe congenital nephrotic syndrome.
- Mutations leading to a reduction of the collagen IV α3α4α5 networks cause Alport syndrome humans, affected individuals have progressive renal disease.

## Key Points 2: Cell-Matrix Adhesion

- Integrins link the extracellular environment to the actin cytoskeleton.
- Integrins do not have intrinsic enzymatic activity; therefore, they recruit effector proteins, which mediate adhesion signaling.
- The laminin receptor integrin α3β1 is the most highly expressed integrin on the podocyte cell surface.
- Homozygous mutations in *ITGA3* in humans lead to congenital nephrotic syndrome.
- The tetraspanin CD151 binds tightly to integrin α3β1 and individuals with mutations in *CD151* develop nephritis.
- Other podocyte cell surface receptors include syndecans and dystroglycan, but the importance of these receptors in the podocyte has yet to be fully determined.

## Key Points 3: Adhesion Complexes

- Adhesion complexes contain over 232 components, which are dependent on cell type and context.
- Talin is an important linkage from integrins to the actin cytoskeleton; podocyte-specific talin 1 knockout mice develop proteinuria and die within 10 weeks.
- Focal adhesion kinase (FAK) is activated in podocytes during glomerular injury.
- Use of FAK inhibitors in mouse models of glomerular disease protects podocyte from injury and the animals from proteinuria.
- The ILK, PINCH, parvin axis is another key linkage from integrins to the actin cytoskeleton.
- Mice, which express ILK, that cannot bind to α-parvin display renal agenesis.
- Mice with podocyte-specific deletion of ILK express GBM defects, loss of slit diaphragms, and podocyte foot process effacement.

## Key Points 4: Cell-Cell Adhesion

- The junction between adjacent podocyte foot processes, termed the slit diaphragm, contains both adherens and tight junction components, in addition to unique components.
- Nephrin and the other NEPH family proteins are key members of this cell junction.
- Mutations in slit diaphrgam proteins cause nephrotic syndrome.
- Slit diaphragm signalling has a major influence on the actin cytoskeleton; adaptors such as NCK1/2, CD2AP, and CRK1/2 are involved in slit diaphragm actin linkage.

## Author Contributions

Rachel Lennon and Michael J. Randles researched the literature for this review; Michael J. Randles prepared the figures; and Rachel Lennon, Michael J. Randles, and Martin J. Humphries contributed to the preparation of the manuscript.

## Conflict of Interest Statement

The authors declare that the research was conducted in the absence of any commercial or financial relationships that could be construed as a potential conflict of interest.
